# Arthritis, or Gout

**Published:** 1838-04-11

**Authors:** N. Chapman

**Affiliations:** Professor of the Theory and Practice of Physic, in the University of Pennsylvania


### MEDICAL EXAMINER.

DEVOTED TO MEDICINE, SURGERY AND THE COLLATERAL SCIENCES.

**EDITED by J. B. BIDDLE, M. D. and M. CLYMER, M. D.**

No. 8. PHILADELPHIA, WEDNESDAY, APRIL 11, 1838. Vol. 1.

### ARTHRITIS, OR GOUT.

*By* N. Chapman, M. D., *Professor of the Theory and Practice of Physic, in the University of Pennsylvania.*

(Concluded from page 107.)

### RETROCEDENT, RETROGRADE, OR MISPLACED GOUT.

Gout, though apparently fixed in one of the extremities, is prone to shift its position, and seize on some internal or vital part, creating, sometimes, the most imminent danger. This is what is denominated retrocedent, or retrograde gout, which may locate itself in any one of the organs of the great cavities, though, most commonly, in the stomach, causing nausea, vomiting, and violent spasms, which, if not expeditiously relieved, will often prove fatal. Gastric affections, of this nature, require a very opposite mode of treatment, in their several stages. It is usual to commence with the diffusible stimulants and antispasmodics, such as opium, ether, musk, carbonate of ammonia, the spirit of turpentine, spiced wine, or ardent spirits, either pure, or converted into hot toddy. The stomach here looses its susceptibility, in a very considerable degree, and hence, in prescribing any of the articles mentioned, the dose must be very much increased. This remark applies, perhaps, with peculiar force, to opium, and its preparations, which may be given in three, or four, or five times the ordinary quantity. As co-operating means, hot fomentations to the epigastric region, are not to be overlooked, in the more vehement of these attacks. They contribute to mitigate pain, and sometimes so far tranquilize the stomach, as to check the puking, and thus prepare the way for the exhibition of medicines.

This is the customary mode of managing the early stage of *retrocedent gout*, in the stomach. Not succeeding, it will be proper to take away some blood. Leeches or cups over the epigastrium, may prove very effectual. Cases, however, occur of such urgency, as not to admit of so slow a process, and venesection must be substituted. Yet, as regards the latter, care and discrimination are to be exercised. The pulse here scarcely can be trusted as a guide, being frequently weak, or depressed, in the more violent attacks, and when perhaps, the loss of blood is imperatively demanded. In making up our decision on this point, we should take into view, the strength of the patient, *the intensity of the pain*, the state of temperature on the surface, and, in fine, the degree of probability, as to the power of the system to react, on depletion.

Notwithstanding all the monitory suggestions against it, by many of the European writers, bleeding, and very copious bleeding too, is habitually directed by me, and other practitioners of this city, in gout of the stomach, and with great success. It is the best, and most certain antispasmodic, and if inflammation exists, holds out the only chance of cure.

There are cases to be managed, essentially in the same manner as common colic. Being spasmodic in the beginning, whatever is calculated to resolve the spasm, will be useful. But phlogosis having taken place, and which sometimes happens, even at an early stage, all stimulating articles are to be abandoned, and measures of a directly opposite tendency resorted to, as general and topical bleeding, and a blister to the epigastrium.

Nearly a similar course should be adopted, when it attacks the bowels, which it is almost as apt to do by violent cramps, and obstinate constipation.

The kidneys are also very liable to be assailed, and with the symptoms of ordinary nephritis, which sometimes may be relieved by fomentation, or the warm bath. But when obstinate, general or topical bleeding, blisters over the affected part, and an opiate, the latter answering best as an enema, are required. Lingering cases, I have found singularly benefited by the use of colchicum. Equally is this treatment suited to an attack of the urinary bladder and of the uterus, which is usually betrayed by severe spasmodic pain.

Translated to the lungs, gout produces a state, sometimes imitative of pneumonic inflammation, and, in other instances, of bronchitis or of asthma. The treatment in each instance is precisely the same, as if the attacks had proceeded from the vicissitudes of weather, or any other of the ordinary causes of such complaints.

Cardiac affections from the same cause, are characterised by violent spasms of the heart, or a state resembling syncope, or by an immediate extinction of life, from suppression of the circulation, or by the slower process of inflammation.

As may be supposed, this affection is calculated always, whether it be violent or not, to create great solicitude and apprehension. Without, indeed, the attack be slight, we are precluded altogether, even from the hope of doing good, by the suddenness of death. Two of the cases which I have met with, terminated almost immediately. Time, however, being allowed to interpose our efforts, venesection and then topical bleeding and blistering constitute the proper means, to be followed up by the use of colchicum or digitalis.

Much of the preceding treatment is equally applicable to gout, when it seizes on the brain. Conforming the practice to the state of the system, we should bleed generally or locally, or both, vesicate the nape of the neck, purge copiously, and do whatever else is called for in other comatose, apoplectic or paralytic conditions, these being the forms which the disease here usually assumes.

In relation to retrocedent gout, there is one precept of universal application, which in practice, must be kept in mind. It is *steadily* to endeavor to invite or restore the disease to the extremities; and, with this view, a pediluvium, made stimulating by the addition of salt, and mustard, or cayenne pepper, and next, sinapisms, or blisters, to the ankles, are the most effectual measures.

### ATONIC, OR IRREGULAR, OR MISPLACED GOUT.

By atonic gout, in the ordinary acceptation of the term, is meant, that state, where, though the arthritic diathesis prevails, there is not vigour enough to induce the inflammatory affection of the limbs, or, if it take place, it is slight or transitory, continuing only for a very short time, and then reverting to some inward structure.

Commonly, the individual is persecuted by a group of affections, which appear chiefly in the stomach, such as loss of appetite, nausea, vomiting, acrid and sour eructations, cardialgia, pyrosis, &c.; with pains and cramps, like those of flatulent cholic, sometimes relieved by a discharge of wind. Constipation usually predominates, though alternated by purging. The urine, as well as the other secretions, is scanty and vitiated, attended with tormina and tenesmus. Connected with this state, the tongue is foul, the skin very dry, the breath heavy and offensive, and great depression of the spirits, amounting to melancholy, often prevails, with a constant and anxious attention to the slightest feelings, much aggravated by a thousand disturbed imaginations and idle fears, to which may be added, palpitations of the heart, or angina pectoris, or asthma, or headache, vertigo, &c. &c. Fever can scarcely be said to exist, though the circulation is occasionally quickened, and irritated.

Considering the articulations as the proper seat of the disease, when it primarily appears in any other part, it receives the title of *misplaced gout.* Disposed to wander about the system, as it is very apt to do, then it is denominated erratic gout, and some instances of it are remarkably distinguished by the suddenness and capriciousness of its fluctuations. Generally incident to an atonic state of the system, we, however, sometimes meet with this variety of it, most decidedly otherwise, or actively inflammatory. *Misplaced gout* of the latter description, may occupy any position, the head, the heart, the lungs, or any one or more of the abdominal viscera. Frequently too, it attacks the testicles, and I knew an elderly man, who had it repeatedly in the penis, causing very painful paroxysms. The skin is, moreover, sometimes assailed by it, manifested by an irritation, resembling prurigo formicans, or by some eruption, particularly on the perinæum or about the anus, or vagina. No part of the body has, in short, an immunity from its attacks, which present every diversity of aspect, simulating all other diseases. Thus occurring, it bears a very close resemblance, to retrocedent gout in the same positions.

The causes of these two varieties of the disease are the same as of regular gout. Men of originally delicate constitutions, or rendered so by depraved indulgences, or in any way, are mainly exposed to such aggressions. Even still more, perhaps, are women, especially after the cessation of the menses, under the guise of some one of those numerous and anomalous affections which they are apt to exhibit at that period.

To discriminate between the real gouty and other conditions, which, though apparently similar, are essentially different, is often a matter of considerable perplexity. Chief reliance must be placed, under circumstances of such embarrassment, on an inquiry into the history of the individual, whether there is reason to suspect an hereditary taint, or from certain habits, it may have been acquired, on a careful comparison of the symptoms themselves, and particularly, on the proneness to metastasis.

Generally, irregular gout is exceedingly troublesome in the management, owing to its disguises, fluctuations, and proteiform shapes. Concentrated, and fixedly in a part, as a phlegmasia, it is least so, though immediately more alarming. But oftener otherwise, it preys upon the constitution, involving organ after organ, till ultimately a general cachexy is induced. The result, on the whole, will depend materially, on the condition of the individual, the integrity of his economy, the simplicity or complication of the case, its duration, the organ or organs affected, and the kind and degree of lesion or lesions.

Of the anatomical characters, little seems to be accurately known. We have, however, abundant evidence though not definitely given, of the existence of spasms, inflammation, acute and chronic, with every sort of disorganization, as the case may have been.

In approaching the pathological consideration, the question which I am first to advert to, is, whether these internal affections are really gout, or of a distinct character superinduced on that peculiar diathesis. Each view may possibly be correct, or, at least, that a totally different affection shall sometimes become thus engrafted. Nevertheless, I think the susceptibility of the internal structures in gout can not be doubted on solid grounds, nor that a very large proportion of such instances are of this nature. Believing, indeed, that both propositions are sufficiently established, I shall dismiss them without further enquiry.

Between misplaced and atonic gout, I take the only, or at all events, the chief difference to consist in the former being more fully developed, and inflammatory, though *in error loci*, while the latter is a feeble, ill defined, and rather an irritative state of the same affection. Even this distinction must be received with limitations. Many of the cases of atonic gout with the aspect of debility, or the loss of tone, I am persuaded, are caused or maintained by chronic phlogosis, or some more serious lesion.

Concerning the treatment of misplaced gout, I have to observe, that we are to be governed by the same principles, and to resort to the remedies, which were detailed in delivering the history of the retrocedent variety of the disease. Let it be here recollected, as a guide to practice, that instead of an inflammatory attack in an extremity, some internal organ is suffering in a similar manner, and requires to be managed accordingly.

It appears very clearly, that in atonic gout, the chylopoietic viscera, are principally, and perhaps it might be affirmed, that they are exclusively the original seat of the disease, those affections remotely distributed, being secondary and dependent on this primordial derangement. By such a view only, am I persuaded, that we can successfully conduct the cure of the generality of these cases. It will sometimes be found, on a careful examination, that chronic gastro-enteritis, alone, or united with functional or other lesions of the liver, is displayed. Can tonics, or cordial stimulants, the customary remedies employed, avail, or rather, would they not be the most inapt or mischievous, which could be selected? Common sense, indeed, dictates an opposite course, or the entire removal of these pathological states, prior to an appeal to any such means. This design having been accomplished, and the restoration of tone seemingly demanded, or in a case originally with this defect only, then may the plan of invigoration be properly entered upon, and with some rational expectation of advantage. But it still more frequently happens, that the chylopoietic organs, without phlogosis, are functionally disordered, showing great depravation in the digestive and nutritive apparatus, resembling ordinary dyspepsia, with its sympathetic disturbances. These several conditions, however, having been amply discussed and disposed of, under the head of the gastric diseases, I shall be content to refer to what was said on that occasion, for the details of the course, as being applicable to the cases now before us. It may, indeed, be enough now to remark, that on the rectification of the organs concerned, by the means formerly mentioned, little else remains to be done in atonic gout, by medicine, than to obviate constipation by magnesia, rhubarb, or sulphur, occasionally directed, and the use of tonics. Of the latter, the chalybeates, alone, or with bark, are much commended. Crude bark, however, I have found to disagree with the stomach, the aromatic decoction or infusion sometimes to answer, and still oftener the tincture, united with one of the martial preparations, though, perhaps, the sulphate of quinine is to be preferred, and certainly as an adjunct to the steel.

Yet there is a writer, who winds up an eulogium on the properties of the bark itself, with the following enthusiastic expression—

"Uno verbo, cortex Peruvianus in podagra, *divinum est remedium.*"

Nearly all of the best authorities, however, seem to unite in denouncing the protracted use of bitters. By invigorating the stomach, they are for a time undoubtedly beneficial—though, a persistence in them, it is alleged, is apt to produce the most pernicious consequences. The power of the system, to throw the disease on the extremities is greatly impaired, and, the individual is rendered miserable, by all the irregular arthritic affections, or he is suddenly destroyed by apoplexy, or has entailed on him palsy, asthma, dropsy, &c. which ultimately prove fatal.

Of the *Portland* powder, you have all heard. This, so called from the duke of Portland, who gave it celebrity, is composed of several of the vegetable bitters, no one of which has much activity; namely, the roots of gentian, and birth-wort, and the tops and leaves of centaury, ground pine, and germander, of equal weights, well dried and pulverised, of which a drachm is to be taken every morning fasting, for months, then omitted for a time, and renewed in less quantity. Yet it is declared by Cullen, by Murray, the author of the "Apparatus Medicaminum," by Darwin, and in short, by many of the best authorities, that this nostrum has proved most hurtful, in the mode I have just mentioned. Exactly the same is said by Galen, and others of the old writers, of nearly a similar compound, which was in vogue in their time. Bitters long continued operate here, by inducing debility of stomach, from over stimulation.

To relieve at the moment, as well as to prevent a recurrence of some of the more harassing affections incident to the disease, as spasms of the alimentary canal, palpitations, &c. &c., I have found the volatile tincture of guiacum, or the carbonate of ammonia, by itself, very serviceable. The Warner's gout cordial, which is an aromatic, carminative tincture of senna and rhubarb, is also deserving of attention. These articles are further calculated to throw the disease on the extremities, an indication never to be lost sight of, or failed to be promoted. By such a translation, when perfect, immediate relief is afforded, however great the previous distress may have been, provided no essential structural lesions have taken place. Directed by this view, a small wine glass full of Warner's cordial especially, should be taken at night, with a copious draught of hot wine whey, a stimulating pedeluvium having been previously used. More active revulsives, as sinapisms or blisters, may also be tried, and I have known in two instances, the wearing of tight boots, to prove effectual.

Not less important than medicines, is a duly regulated regimen, concerning which, however, I have little to add, to what was said on this point, in regard to the stomach affections. It differs chiefly, in the food requiring, in some instances, to be more stimulating, and in a free use of wine.

The gout having been allured to a joint, it will hardly be prudent to attempt any means to lessen the pain or inflammation. But on the contrary, our aim should be, so to establish it, as to let it pass through all the stages regularly, without the least molestation. This is a case which may be properly committed "to flannel, and patient endurance," with the exception, that should it shew a disposition to recede, such an event must be obviated by pretty much the same means employed to bring it to the extremity. We may, however, be compelled to direct wine itself, and more liberally, and also, to resort to opiates, or the carbonate of ammonia, or musk, which last article, though less prescribed, is, when of good quality, one of the most efficient remedies with which I am conversant.

Closing this part of my subject, I cannot forbear to urge the value of our mineral and thermal springs, in their relations to every modification of gout. Those of Europe of a similar kind, have immemorially had an indisputable reputation in this respect, and to which I have reason to believe our own are still more entitled, from greater efficacy. The waters of Bedford in this state, of Saratoga in New York, and of the White Sulphur Springs in Virginia, are eminently calculated to repair the derangements of the primæ viæ, the liver, and kidneys, incident to the disease, and the warm and hot baths in the same neighbourhood as the last, are not less so, to re-establish a healthy condition of the skin, very frequently dry and harsh, with a feeble capillary circulation, to invigorate the nervous system, nearly always out of order, and above all, perhaps, are they serviceable in the cure of muscular weakness of the lower limbs, and the chronic swellings, rigidities and other injuries of the articulations. The first is to be preferred in reference to the general affections named, and the second, to relieve the topical lesions, especially, when applied as douches, followed by frictions and shampooring. Nothing need be said of the influence of the long journey to reach these springs, or of the delicious climate of the locations, or of the charms of the society by which they are distinguished.

Though the complete eradication of gout, in any of its forms, when fully established, is perhaps hardly to be expected, still, by care in the avoidance of the exciting causes, the interval between the paroxysms may be procrastinated, and when they occur, greatly abated. Cullen's directions on this point are so sound, that I cannot, forbear to recommend them to your perusal, and most particular regard.

As indispensable, let me insist on a strict undeviating adherence to a moderate diet. Medical authority, however, differs as to the kind of food. By Redi, Starke, Lobb, &c. the exclusive use of vegetables is strenuously advised, while Brown and his disciples, as strongly urge its being the reverse. Both are wrong. The lowest, and most abstemious diet will sometimes be exacted, and on other occasions, the cordial, and generous. Limitation in quantity, is, perhaps, of as much consequence as the quality of the nutriment. Discrimination is no less to be made in relation to drinks. To some, water alone should be allowed, and others will require a small portion of wine, or of ardent spirits. The great purpose is to preserve soundness of the digestive, and assimilative functions, and the course of living must be accommodated accordingly.

Equally important is it, that habits of indolence, or even of too intense application to study, or any other sedentary occupation, be exchanged for those of activity and exercise. Many, says Hoffman, "have lost their gout, with their fortune," by turning, no doubt, at the same time, from luxury, and lazy enjoyments to laborious pursuits. The eccentric Mr. Abernethey being asked by a nobleman, what would prevent gout, replied in his usual sententious manner: My Lord: "Live on sixpence a day and earn it." These are aphorisms, which, though uttered with some exaggeration, contain a vast deal of wisdom, and might be adopted, with proper qualifications, as a rule of life, in reference to the object in view.

By one of the sages of our profession, gout has been pronounced as proceeding entirely, from "vexation of spirit." Not agreeing entirely with him, the immense influence of moral causes in the origination and maintainance of the disease, seems to be universally admitted. Every emotion or passion, whether of tempestuous excitement, or the reverse, depressive, or worrying and irritative, has such an effect. Equanimity is hence to be cultivated, and which will be most successfully done, by resorting to those sentinels, which philosophy places over us, to protect against the incursions of the evil dispositions by which the soul becomes agitated or disturbed. Nourished by unresistance, a fretted or exasperated temper is held to be especially baneful, and hence, above all, the injunction of the wisest of mortals, should be remembered: "Let not the sun go down on thy wrath."

Yet, admonished as they may be, we shall find our patients, for the most part, reckless of advice; and, like those of the divine, or moralist, our preachings, too often, leave only an evanescent impression. The victims of gout, are also usually the votaries of sensual gratifications. By the ancients, who under the shadow of an allegory, often conveyed a truth, the disease was held to be the progeny of the divinities of love and debauchery. To such persons, as I have alluded to, we shall in vain hold the language of remonstrance, or deliver injunctions to reformation, when opposed by the attractions of the festive board, the seductions of luxurious ease, and the other fascinations and enjoyments of sense and appetite. Ease, in such cases, retracts the vows made in pain, and exclaims with the voluptuous poet of antiquity, "*Vitam faciunt balnea, vina, venus.*"

It remains only to add, that, in gout, the paroxysm may be sometimes warded off, if, on the first signs of its approach, a purge be taken. The same good effect I have also known from repeated doses of the alkalies, or magnesia, the prepared oyster-shell, or other antacids, and also, by the use of colchicum.

An allusion has already been made to the denunciation of the latter article under any circumstances, and more especially, to any thing like an habitual employment of it. But these apprehensions, I must think, are unfounded, and certainly to the extent to which they have been expressed, by some of the European writers. Much as I have prescribed it, never have I known any pernicious effects from it, and, I believe, that it may be always so regulated, as to prove a perfectly safe remedy. There are within the compass of my own experience several individuals who were formerly harassed by floating gout, or frequently repeated regular attacks of it, from which their constitution suffered materially, that have obviated the paroxysms, and greatly improved in health, by a pretty constant resort to this medicine, even for a term of years. It is generally taken in the small dose of ten or twenty drops daily, morning and evening, when any slight indications of gout exist, increasing the quantity considerably, on a stronger manifestation of an attack.

In recurring to the whole of what has been delivered, you will perceive, that I have suggested little very peculiar in the management of gout. Too long, indeed, have we been accustomed to view it with a sort of superstitious awe. We have been afraid to touch it. Ever since the time of Sydenham, with some few exceptions, the approach of practitioners to this disease, has been singularly marked by timidity, and encumbered with doubt and irresolution. The first step towards a correct investigation of its pathology, and, of course, a more successful practice, is, to discard such apprehensions, and to contemplate it pretty much as ordinary disease, to be cured for the most part, by common remedies.

